# Evaluation of Health-Promoting Behaviors in the Prevention of Cardiovascular Diseases in the Preschool Children of Polish Health Care Professionals

**DOI:** 10.3390/ijerph19010308

**Published:** 2021-12-28

**Authors:** Marta Gruca, Justyna Zamojska, Katarzyna Niewiadomska-Jarosik, Agnieszka Wosiak, Elżbieta Smolewska

**Affiliations:** 1 Department of Pediatric Cardiology and Rheumatology, Medical University of Lodz, Sporna 36/50, 91-738 Lodz, Poland; j.zamojska@wp.pl (J.Z.); kasiajarosik@wp.pl (K.N.-J.); elzbieta.smolewska@umed.lodz.pl (E.S.); 2 Institute of Information Technology, Lodz University of Technology, 91-738 Lodz, Poland; agnieszka.wosiak@p.lodz.pl

**Keywords:** cardiovascular diseases, children, social determinants of health

## Abstract

Background: The aim of the study was to evaluate the health-promoting behavior of the preschool children (aged 3–6 y) of Polish health care professionals (HCPs). Methods: The study was conducted by means of quantitative research on a group of 386 individuals, using an Internet-based survey. Results: The ideal cardiovascular health model was determined in the case of 22 children (5.6%). The collected data revealed that, when regarding the recommended level of physical activity, children from HCP families meet the American Heart Association criteria much more often than their peers from other study populations (56.5% vs. 16.6%). In our study, more girls than boys participated in organized activities (60.2% vs. 50.3%, *p* = 0.05). There was no correlation between achieving adequate levels of physical activity and the BMI (*p* > 0.1). Overweight children had a more balanced diet than children with a normal body weight (*p* = 0.009). Conclusions: The obtained results allowed us to make the conclusion that there is a need to implement educational and preventive measures on a large scale, while some health-promoting behaviors, especially those concerning proper nutrition, require major modifications, even in HCP families.

## 1. Introduction

Cardiovascular diseases (CVDs) and their complications are the main cause of work disability in developed countries. According to the data provided by the Central Statistical Office, CVDs are responsible for 46% of deaths in the adult population of Poland [1]. The development of CVDs in the adult population is highly correlated with cardiovascular risk factors that may first occur in childhood and then progress towards more severe forms [2]. Despite the fact that the clinical symptoms of CVDs and the associated complications rarely manifest themselves in children, it should be remembered that even preschool children are exposed to CVD risk factors [3,4]. The prevention of cardiovascular diseases is defined as a coordinated set of actions to be taken at the population level, or targeted at individuals, for the purpose of eliminating or minimizing the consequences of CVDs and the associated disabilities [5]. The effectiveness of such preventive actions has been proven by numerous population studies, including the CARDIA study [6]. In the Polish adult population, the problem of increasing exposure to CVD risk factors was analyzed as a part of nationwide studies, such as NATPOL PLUS, PolSenior, and WOBASZ [7,8,9]. A great source of information regarding the health status of the developing population is the OLAF and OLA studies, which provided the basis for establishing new growth charts for Polish children. The Mother’s and Child Institute in Warsaw published a report that focuses on the assessment of physical activity in the developing population [10]. A number of studies have been carried out to assess the prevalence of overweight and obesity among preschool children both in Poland and in neighboring countries [11,12,13]. The ToyBox cross-sectional study compared levels of energy-balance-related behaviors (physical activity, sedentary behavior, and dietary behaviors in four- to six-year-old preschoolers from six European countries (Belgium, Bulgaria, Germany, Greece, Poland, and Spain) [14]. Nevertheless, there is a paucity of research in Poland focusing on complex CVD prevention in children. As regards health-promoting behaviors, the least studied age group is preschool children. The following publication analyzes the health-promoting behaviors of preschool children raised in HCP families in the context of their cardiovascular health condition. Our study focuses on four health-promoting factors or behaviors, i.e., no exposure to tobacco smoke, a normal BMI, an adequate physical activity level, and a healthy diet. Considering knowledge on cardiovascular health possessed by healthcare professionals, it should be assumed that children growing up in HCP families will be provided with an ideal cardiovascular health model.

## 2. Materials and Methods

### 2.1. Participants

The analysis included 386 respondents; the target group was mothers who were HCPs (medical practitioners). A purposive sampling method was used in our study. The study was conducted by means of a quantitative research, using an Internet-based survey. The questionnaire was accessible on a closed online forum only for mothers who are HCPs. Participation in the study was voluntary. Through the anonymous questionnaire, the protection of respondents’ privacy was achieved and psychological harm was avoided. The questions in the questionnaire did not contain sensitive content. The submission of the completed questionnaire was considered consent to participate in the survey; therefore, the approval of the bioethics committee to conduct the survey was not required. The questionnaire assessed the health-promoting behavior of preschool children (no exposure to tobacco smoke, appropriate BMI, appropriate level of physical activity, and healthy diet). There were no missing values in the data gathered and no outliers to handle separately. The characteristics of the group are presented in Table 1 and Table 2.

According to the AHA report, the definition of an ideal cardiovascular health model means no exposure to tobacco smoke, the right BMI, adequate levels of physical activity, and a healthy diet [15]. The BMI was calculated based on the data provided in the questionnaire (i.e., the child’s body mass and height). The ideal BMI according to the AHA report is BMI < 85th percentile. Underweight is defined as BMI < 5th percentile, overweight as a BMI between 85th and 95th percentile, whereas obesity as BMI > 95th percentile, appropriate for the sex and age. The survey study also assessed the children’s exposure to tobacco smoke, their physical activity, and their dietary habits. A healthy diet was understood as the daily consumption of vegetables and fruit, eating fish at least twice a week, and eliminating sugary drinks. In addition, the respondents were asked how their children usually spent their free time at home. That questions were adopted from a questionnaire used in the report by Fijałkowska et al. [10]. Our results were compared with those presented in other population studies [10,11,15,16,17].

### 2.2. Statistical Analysis

The analyzed parameters were presented as the basic measures of the structure description: the mean with the standard deviation (SD), medians, and structure and intensity indicators. The analyzed parameters were examined in terms of differences existing between the groups. These differences were checked for statistical significance. The statistical significance threshold was assumed to be *p* ≤ 0.05. In order to verify differences between the two groups, the Student’s *t*-test (for variables consistent with a normal distribution) and Mann–Whitney’s non-parametric U-test were used. For testing the differences among more than two groups, the ANOVA single-factor analysis of variance (for variables consistent with a normal distribution) and the Kruskal–Wallis test (for non-parametric variables) were used. Categorical variables were compared with a chi-squared or Fischer’s test. The verification of a normal distribution was performed using the Shapiro–Wilk test. The relationship between the selected parameters was analyzed by calculating Spearman’s rank correlation coefficient. The assessment of the differential effect of the variables on the selected groups was performed based on a discriminatory analysis using Wilks’ Lambda measure. The statistical analysis was carried out using Statistica 13 (StatSoft).

## 3. Results

The data collected via the Internet questionnaire concerned 386 children (191 girls and 195 boys) of preschool age. In the study group, a significantly higher number of three-year-olds (33.4%) was recorded as compared to six-year-olds (15.0%) (*p* < 0.001) (Table 1).

Based on the provided information about the children’s health-promoting behaviors, the ideal cardiovascular model was identified in only 22 children (5.6% of all respondents). They were children not exposed to passive smoking, with BMI < 85th percentile, physically active for about one hour a day, and eating a healthy diet. The analysis revealed that the largest group of children had an appropriate BMI (290 children, i.e., 75.1%), and the differences within group were statistically significant (*p* < 0.001). The group included 147 girls (77.0% of all the girls) and 143 boys (73.3% of all the boys). Overweight was determined in twenty-five children (6.5%)—ten girls (5.2% of all the girls) and fifteen boys (7.7% of all the boys). Obesity was identified in the case of seven boys (3.6% of all boys), and in this group, the difference between the sexes was statistically significant (*p* = 0.01). The ideal BMI was calculated for 354 children (91%): it was observed that the girls much more frequently (94.8% of all the girls) had the ideal BMI compared to the boys (88.7% of all the boys). The differences between the sexes were statistically significant (*p* = 0.03). In the study group, there was no correlation between age and BMI value (*p* = 0.16).

Eight children (2.0%) were exposed to tobacco smoke in their immediate environment; however, the smokers were either their grandparents or nannies, not their parents.

A well-balanced diet was determined in 36 children (9.3% of all the children). There was no statistically significant difference in a properly balanced diet in terms of sex or age. Everyday fruit consumption was observed in 277 children (71.8%), whereas vegetables were eaten daily by 257 (66.6%) children. Eighty children (20.7%) ate sweets every day; only seven children (1.8%) were not given any sweets at all; thirty-seven children (9.6%) had sweets occasionally, less often than once a week. Two-hundred and forty-six children (63.7%) did not drink sweetened beverages at all. Fish consumption by the children raised in HCP families was relatively low. Only 73 children (18.9%) ate fish at least twice a week, and 204 children (52.8%) had fish once a week. The diet of 17 children (4.4%) did not include any fish at all. Statistically, more boys did not eat fish at all as compared to the girls (6.8% vs. 2.1%; *p* = 0.03).

Physical activity > 60 min, 7 d a week, was reported in the case of 218 children (56.5%), including 120 boys (61.5%) and 98 girls (51.3%), and the difference was statistically significant (*p* = 0.04). Two-hundred and thirteen children (55.2%) participated in organized activities for 2 h a week, on average, and the difference was also statistically significant (*p* = 0.05). More girls than boys participated in organized activities (60.2% vs. 50.3%, *p* = 0.05). Undoubtedly, the highest percentage of six-year-olds (86.2% of all the six-year-old children) took part in organized forms of activity. The difference between the individual age groups was statistically significant (Table 3).

More girls had an ideal BMI and participated in organized forms of activity, and the appropriate level of physical activity was more frequently reached by boys than girls (Table 4).

The most popular forms of physical activity mentioned by the respondents included swimming (25.0%), football (19.0%), dancing (13.0%), gymnastics (10.0%), cycling (7.0%), and martial arts (7%).

As regards spending free time at home, most of the children were constantly on the move (51.6%) or displayed “mixed” behaviors (47.2%), i.e., dividing their free time between active and passive leisure. Only five children (1.3%) preferred more quiet activities (*p* < 0.001). A correlation was found between the type of time spent at home and achieving the proper level of physical activity. Statistically, more children who, when spending their time at home were constantly on the move, took up sufficient physical activity as compared to the other two groups (*p* < 0.001) (Table 5).

## 4. Discussion

According to the AHA publication, there is a need for the primary prevention of CVDs already in the pediatric population [15]. The AHA introduced a scale for determining the presence of four health-promoting factors or behaviors, i.e., no exposure to tobacco smoke, appropriate BMI, appropriate level of physical activity, and healthy diet (4–5 meals eaten at regular hours, limited amount of salt and sugar in the diet, eating vegetables, fruit, fish, and whole-grain products) and three health indicators (correct total cholesterol level, blood pressure, and fasting blood glucose level) [15]. In the AHA report, children aged 2–5 y were assessed only in terms of their BMI. Other factors, such as exposure to tobacco smoke, dietary habits, or physical activity, were not included in the analysis. Based on the AHA report, 78–80% of children aged 2–5 y were found to have an ideal BMI. According to the AHA report, girls demonstrated a higher prevalence of poor BMI (≥95th percentile) status as compared to boys. In our report, only the boys were overweight, and in this group, the difference between the sexes was statistically significant (*p* = 0.01).

In Poland, Kułaga et al. conducted a study involving a group of 5026 children aged 2–6 y. The results revealed excessive body mass in 12.2% of the boys and 10% of the girls, while 4.9% of the boys and 3.4% of the girls of preschool age were obese [16]. In our analysis, the percentage of overweight boys and girls was slightly lower (7.7% vs. 12.20% and 5.2% vs. 10.0%, respectively). A similar percentage of the boys was obese in both studies (3.6% vs. 4.9%); none of the girls participating in our research were obese.

Furthermore, Matłosz et al. assessed the prevalence of overweight and obesity among Polish children aged 5–6 y. In this age group in boys, 6.5% were overweight and 3.8% were obese. Likewise, in girls, 6.8% were overweight and 4.9% were obese. The authors found a non-significant trend in the prevalence of overweight and obesity, defined according to the BMI, in both genders [11].

The four health-promoting behaviors include a lack of exposure to tobacco smoke. In the case of preschool children, we considered exposure to passive smoking, since it has been proven to have the same negative effects on health as active smoking. Exposure to passive smoking is associated with frequent respiratory tract infections, as well as progression in the frequency and severity of asthma symptoms. Exposure to tobacco smoke in early childhood results in chronic cardiovascular changes that become clinically evident in adulthood [17,18]. Despite multiple educational campaigns, one in two Polish children is exposed to the toxic effects of tobacco smoke. According to the results of a nationwide Polish survey on attitudes toward tobacco smoking, as many as 14% of Polish adults are exposed to passive smoking in public spaces. In 2017, 5% of Polish adults were exposed to passive smoking in children’s playgrounds. Comparing the results of the recent report to data from previous years, it seems that most smokers still smoke in the presence of non-smoking persons. Nearly every fifth Polish adult admits to smoking in the presence of children. Tobacco smokers live in up to 40% of Polish households [19]. In the analyzed group, fewer children (only 3%) were exposed to passive smoking as compared to the data presented above. It is disturbing that despite medical knowledge of the harmfulness of passive smoking, children from HCP families are exposed to tobacco smoke in their home environment. However, we do not know how many children participating in the survey were exposed to passive smoking in public spaces.

Behaviors having a positive influence on the functioning of the cardiovascular system include regular, appropriate physical activity that, in the case of adults, reduces general mortality and CVD morbidity by 20–30% [20,21]. Family relationships have a huge influence on the formation of health-promoting attitudes among children [22]. It has been proven that physical activity habits established in childhood are continued in adulthood [23,24]. Hence, ensuring an appropriate level of physical activity is crucial even in early childhood. Tremblay et al. specified the amount of physical effort recommended for preschool children. According to the researchers, children aged 3–4 y should be physically active for at least 180 min a day. Such physical exercise should include at least 60 min of vigorous play. Children aged over 5 y should spend at least 60 min a day engaged in mild to intense physical activities. Examples of mild physical activities include brisk walks, hiking, and catch-and-throw games (e.g., baseball). Sports requiring more intense physical effort include running, cycling, and running and catching games (e.g., football). Intense physical activity should be undertaken at least thrice a week [25]. Children of preschool age should be provided as many opportunities to spend active time as possible, and parents should not limit their physical activity, as long as it is safe [26,27]. As compared to the group of preschool children taking part in the report by Fijałkowska et al., the children whose mothers were HCPs more often participated in organized physical activities (55.2% vs. 47.2%) and were more active when staying at home (51.6% vs. 27%). Both children from HCP families and their peers in the aforementioned study chose swimming from among the organized forms of activity [10]. The collected data revealed that, regarding the recommended level of physical activity, children from HCP families meet the AHA criteria much more often than their peers (56.5% vs. 16.6%) [10]. According to the data from the Mother and Child Institute, the percentage of children of preschool age who performed the AHA-recommended amount of physical activity may be underestimated. In the publication by Fijałkowska et al., it was suggested that parents might not consider certain forms of activity as playing, hence the considerable difference in the reported amounts of physical activity [10]. It seems that participation in organized activities helps to achieve the correct level of physical activity. This study did not confirm this correlation, neither in our study group, nor in the group of children from Fijałkowska et al.’s report. Both studies proved that the type of daily household activity strongly correlates with the achievement of the recommended level of physical activity in a usual week *p* < 0.001 (Table 4). In our study, there was no correlation between achieving adequate levels of physical activity and the BMI (Table 6).

A balanced diet, followed from an early childhood, is believed to be very beneficial for health [28]. It decreases the risk of CVDs by preventing obesity, hypertension, and diabetes [2]. The AHA has created a recommendation for a well-balanced diet among children with regard to cardiovascular health [15]. There are no such recommendations for the pediatric European population. The European Society of Cardiology focuses on CVD prevention in the adult population [29,30]. Therefore, in this article, we referred to the AHA studies. Despite the fact that the AHA has performed a very comprehensive study of dietary habits in the pediatric population, it has also not evaluated preschool children. The AHA report reveals that around 91% of American children have an ill-balanced diet and the ideal nutrition model as per the recommendations was found in only 0.5% of children [15]. The AHA study did not include preschool children. Our study revealed that, despite the widespread knowledge of healthy eating, only 36 children from the HCP families (9.3%) had a well-balanced, healthy diet. The obtained results lead to the conclusion that the greatest problems are a too low consumption of fish and the presence of sweets in the diet of preschool children. The consumption of fish according to the AHA recommendations was declared in only 18.4% of children. With regard to sweets, only 1.8% of children did not eat them at all, while 9.6% of children were given sweets occasionally, less often than once a week. Daily fruit consumption was reported in the case of 277 children (71.8%), and 257 children (66.6%) had vegetables in the diet every day. A valuable source of information on the health behavior of preschool children was provided by the ToyBox cross-sectional study. The aim of the above study was to compare the levels of energy balance behaviors (physical activity, sedentary lifestyle, eating behaviors (especially drinking water, consumption of sugar-sweetened drinks, and unhealthy snacking) in four- and six-year-old preschoolers from six European countries (Belgium, Bulgaria, Germany, Greece, Poland, and Spain). Based on the compiled results, Bulgaria and Greece reported lower levels of physical activity and higher levels of sedentary behavior compared to the other countries participating in the study. Belgium, Germany, and Poland reported lower water consumption and higher consumption of sugar-sweetened beverages and snacking. In addition, a low proportion of preschool children met the recommendations for physical activity, sedentary behavior, and water intake [14].

## 5. Conclusions

The discriminatory analysis and the Shapiro–Wilk test did not show any impact of eating patterns on physical activity in our survey. Based on all the data obtained, it may be concluded that the children from HCP families, despite noticeable diet mistakes, more often than their peers from other studies, fulfilled the criterion of appropriate physical activity.

Our study revealed that health-promoting behaviors are more often observed in children from HCP families than in their peers from other population studies [8,9,10]. Our analysis demonstrates a relationship between balanced nutrition and the BMI. Overweight children have a better balanced diet than children with a normal body weight. It can be assumed that parents see the problem of overweight in their children and attach more importance to a balanced diet to reduce their children’s weight (Table 7).

Even so, the dietary habits of preschool children (including those raised in HCP families) should be improved, their exposure to passive smoking should be eliminated, and they should be encouraged to be more physically active. The obtained results allow us to make the conclusion that there is a need for the implementation of educational and preventive measures on a large scale, while some health-promoting behaviors, especially those concerning proper nutrition, require major modifications, even in HCP families. The prevention of cardiovascular diseases should be based on teaching the youngest children the optimal, healthy lifestyle. This would support them in continuing to follow that model also as adults, thus avoiding or minimizing the risk of CVDs and the associated complications. Nonetheless, there is still a need for additional study. Therefore, further research is planned to investigate future interventions to reveal new correlations between demographic and intervention data. The limitations of this study arise from the use of an online survey as a research method. Another limitation might also be the subjective parental report about the dietary habits of preschool children. The study group was not representative of the Polish population, as the target group was children of health care professionals. The results suggest that a study evaluating health-promoting behavior should be conducted among a larger group of respondents.

## Figures and Tables

**Table 1:** Baseline characteristics of the study group.

Parameter		Studied Group (N = 386)	Percentage
Sex (F/M)	F	191	49.48%
	M	195	50.52%
Age (years)	3	129	33.42%
	4	105	27.20%
	5	94	24.35%
	6	58	15.03%
BMI	Underweight	64	16.58%
	Appropriate body mass	290	75.13%
	Overweight	25	6.48%
	Obesity	7	1.18%
Smoking parents	No	374	96.89%
	Yes	12	
Smoking next to the child	Not applicable	374	96.89%
	No	12	3.11%
	Yes	0	0%
Passive smoking	No	378	97.93%
	Yes	8	2.07%
Physical activity at home	When playing, the child displays “mixed” behaviors, i.e., dividing their free time between active and passive leisure	182	47.15%
	The child is always on the move	199	51.55%
	The child usually plays in a sitting position, watches TV, colors drawings, etc.	5	1.30%
Organized forms of activity	No	173	44.82%
	Yes	213	55.18%

**Table 2:** Characteristics of the study group by gender.

Parameter	F (N = 191)		M (N = 195)		*p*
	Mean Value	SD	Mean Value	SD	
Height (m)	1.08	0.09	1.08	0.09	0.70
Body mass (kg)	17.44	3.54	18.04	3.53	0.10
BMI (kg/m^2^)	14.85	1.36	15.28	1.64	0.005

**Table 3:** Characteristics of the study group by age.

Parameter		Age = 3 (N = 129)		Age = 4 (N = 105)		Age = 5 (N = 94)		Age = 6 (N = 58)		*p*
		N	Percentage	N	Percentage	N	Percentage	N	Percentage	
Gender	F	71	55.04	49	46.67	44	46.81	27	46.55	0.50
	M	58	44.96	56	53.33	50	53.19	31	53.45	
BMI	Underweight	27	20.93	15	14.29	13	13.83	9	15.52	0.79
	Appropriate body mass	95	73.64	81	77.14	70	74.47	44	75.86	
	Overweight	6	4.65	7	6.67	8	8.51	4	6.90	
	Obesity	1	0.78	2	1.90	3	3.19	1	1.72	
Physical activity at home	When playing, the child displays “mixed” behaviors, i.e., dividing their free time between active and passive leisure	61	47.29	42	40.00	49	52.13	30	51.72	0.61
	The child is always on the move	68	52.71	61	58.10	43	45.74	27	46.55	
	The child usually plays in a sitting position, watches TV, colors drawings, etc.	0	0.00	2	1.90	2	2.13	1	1.72	
Organized forms of activity	No	83	64.34	51	48.57	31	32.98	8	13.79	<0.001
	Yes	46	35.66	54	51.43	63	67.02	50	86.21	

**Table 4:** Health model and its components with stratification according to gender.

Parameter	Total (N = 386)				F (N = 191)				M (N = 195)				*p*-Value for Differences between Genders
	Yes	%	No	%	Yes	%	No	%	Yes	%	No	%	
Ideal health model	22	5.67	364	94.30	9	4.71	182	95.29	13	6.67	182	93.33	0.41
Ideal BMI (<85 percentile)	354	91.71	32	8.29	181	94.76	10	5.24	173	88.72	22	11.28	0.03
Balanced diet	36	9.33	350	90.67	17	8.90	174	91.1	19	9.74	176	90.26	0.78
Physical activity 7 days a week > 60 min	218	56.48	168	43.52	98	51.31	93	48.69	120	61.54	75	38.46	0.04
Organized forms of activity	213	55.18	173	44.81	115	60.21	76	39.79	98	50.26	97	49.74	0.05

**Table 5:** The relationship between the type of time spent at home and taking up sufficient physical activity.

Parameter	When Playing, the Child Displays “Mixed” Behaviors, i.e., Dividing Their Free Time between Active and Passive Leisure (N = 182)		The Child Is Always on the Move (N = 199)		The Child Usually Plays in a Sitting Position, Watches TV, Colors Drawings, etc. (N = 5)		*p*-Value
	N	%	N	%	N	%	
Physical activity 7 days a week > 60 min = No	95	52.20	69	34.67	4	80	<0.001
Physical activity 7 days a week > 60 min = Yes	87	47.80	130	65.33	1	20	

**Table 6:** The relationship between the appropriate level of physical activity and the BMI.

Parameter	Physical Activity 7 Days a Week > 60 min = No (N = 168)		Physical Activity 7 Days a Week > 60 min = Yes (N = 218)		*p*-Value
	N	%	N	%	
Underweight	29	17.26	35	16.06	0.95
Appropriate body mass	125	74.40	165	75.69	
Overweight	11	6.55	14	6.42	
Obesity	3	1.79	4	1.83	

**Table 7:** The relationship between a well-balanced diet and body mass.

	Balanced Diet = No (N = 350)		Balanced Diet = Yes (N = 36)		*p*-Value
	N	%	N	%	
Underweight	59	16.86	5	13.89	<0.001
Appropriate body mass	265	75.71	25	69.44	
Overweight	19	5.43	6	16.67	
Obesity	7	2.00	0	0.00	

## Data Availability

The data used to support the findings of this study are included within the article.

## References

[1] Gus Rocznik Statystyczny Rzeczypospolitej Polskiej 2016. Statistical Yearbook of the Republic of Poland 2016. https://stat.gov.pl/obszary-tematyczne/roczniki-statystyczne/roczniki-statystyczne/rocznik-statystyczny-rzeczypospolitej-polskiej-2016,2,16.html.

[2] Bouhanick B., Sosner P., Brochard K., Mounier-Véhier C., Plu-Bureau G., Hascoet S., Ranchin B., Pietrement C., Martinerie L., Boivin J.M. (2021). Hypertension in Children and Adolescents: A Position Statement from a Panel of Multidisciplinary Experts Coordinated by the French Society of Hypertension. Front. Pediatrics.

[3] Williams C.L., Strobino B.A., Bollella M., Brotanek J. (2004). Cardiovascular Risk Reduction in Preschool Children: The “Healthy Start” Project. J. Am. Coll. Nutr..

[4] Yin L., Wills H., Clarke N., Shacks J., Bottrell C., Poulsen M.K. (2009). Cardiovascular Risk in Preschool Children. ICAN Infant Child Adolesc. Nutr..

[5] Porta M. (2014). A Dictionary of Epidemiology.

[6] Liu K., Daviglus M.L., Loria C.M., Colangelo L.A., Spring B., Moller A.C., Lloyd-Jones D.M. (2012). Healthy Lifestyle through Young Adulthood and the Presence of Low Cardiovascular Disease Risk Profile in Middle Age. Circulation.

[7] Bledowski P., Mossakowska M., Chudek J., Grodzicki T., Milewicz A., Szybalska A., Wieczorowska-Tobis K., Wiecek A., Bartoszek A., Dabrowski A. (2011). Medical, Psychological and Socioeconomic Aspects of Aging in Poland. Exp. Gerontol..

[8] Broda G., Rywik S. (2005). Wieloośrodkowe ogólnopolskie badanie stanu zdrowia ludności--projekt WOBASZ. Zdefiniowanie problemu oraz cele badania. Multicenter national Polish population health status tests—WOBASZ project with defined problems and treatment goals. Kardiol. Pol..

[9] Zdrojewski T., Bandosz P., Szpakowski P., Konarski R., Manikowski A., Wołkiewicz E., Jakubowski Z., Łysiak-Szydłowska W., Bautembach S., Wyrzykowski B. (2004). Rozpowszechnienie głównych czynników ryzyka chorób układu sercowo-naczyniowego w Polsce. Wyniki badania NATPOL PLUS. Prevalence of main risk factors of cardiovascular diseases in Poland. Results of the NATPOL PLUS study. Kardiol. Pol..

[10] Fijałkowska A., Mazur J., Oblacińska A., Nałęcz H., Jodkowska M., Korzycka M., Kolipińska E., Dzielska A., Kleszczewska D., Radiukiewicz K., Fijałkowska A. (2018). Aktualna Ocena Poziomu Aktywności Fizycznej Dzieci i Młodzieży w Wieku 3–19 Lat w Polsce. [Assessment of the Level of Physical Activity of Children and Youth Aged 3–19 in Poland].

[11] Matłosz P., Wyszyńska J., Asif M., Szybisty A., Aslam M., Mazur A., Herbert J. (2021). Prevalence of Overweight, Obesity, Abdominal Obesity, and Obesity-Related Risk Factors in Polish Preschool Children: A Cross-Sectional Study. J. Clin. Med..

[12] Sedlak P., Pařízková J., Procházková L., Cvrčková L., Dvořáková H. (2017). Secular Changes of Adiposity in Czech Children Aged from 3 to 6 Years: Latent Obesity in Pre-school Age. BioMed. Res. Int..

[13] Żegleń M., Kryst Ł., Kowal M., Woronkowicz A., Sobiecki J. (2020). Changes in the Prevalence of Overweight/Obesity and Adiposity among Pre-School Children in Kraków, Poland, from 2008 to 2018. J. Biosoc. Sci..

[14] De Craemer M., Lateva M., Iotova V., De Decker E., Verloigne M., De Bourdeaudhuij I., Androutsos O., Socha P., Kulaga Z., Moreno L. (2015). Differences in Energy Balance-Related Behaviours in European Preschool Children: The ToyBox-Study. PLoS ONE.

[15] Steinberger J., Daniels S., Hagberg N., Isasi C., Kelly A., Lloyd-Jones D., Pate R., Pratt C., Shay C., Towbin J. (2016). Cardiovascular Health Promotion in Children: Challenges and Opportunities for 2020 and Beyond: A Scientific Statement from the American Heart Association. Circulation.

[16] Kułaga Z., Gurzkowska B., Grajda A., Wojtyło M., Góźdź M., Litwin M. (2016). The prevalence of overweight and obesity among Polish pre-school-aged children. Dev. Period Med..

[17] Burroughs Peña M.S., Swett K., Kaplan R.C., Perreira K., Daviglus M., Kansal M.M., Cai J., Giachello A.L., Gellman M.D., Velazquez E.J. (2018). Childhood and Adult Exposure to Secondhand Tobacco Smoke and Cardiac Structure and Function: Results from Echo-Sol. Open Heart.

[18] Echouffo-Tcheugui J.B., Erqou S., Butler J., Yancy C.W., Fonarow G.C. (2016). Assessing the Risk of Progression from Asymptomatic Left Ventricular Dysfunction to Overt Heart Failure. JACC Heart Fail..

[19] Raport z Ogólnopolskiego Badania Ankietowego na Temat Postaw Wobec Palenia Tytoniu. Kantar dla Głównego Inspektoratu Sanitarnego. [Report from a Nationwide Survey on Attitudes towards Smoking. Kantar Public for the Chief Sanitary Inspectorate]. https://gis.gov.pl/wp-content/uploads/2018/04/Postawy-Polak%C3%B3w-do-palenia-tytoniu_Raport-Kantar-Public-dla-GIS_2019.pdf.

[20] Tian D., Meng J. (2019). Exercise for Prevention and Relief of Cardiovascular Disease: Prognoses, Mechanisms, and Approaches. Oxidative Med. Cell. Longev..

[21] Sattelmair J., Pertman J., Ding E.L., Kohl H.W., Haskell W., Lee I.-M. (2011). Dose Response between Physical Activity and Risk of Coronary Heart Disease. Circulation.

[22] Moral-García J.E., Urchaga J.D., López-García S. (2021). Relevant Factors in Adolescent Well-Being: Family and Parental Relationships. Int. J. Environ. Res. Public Health.

[23] Woods C.B., Volf K., Kelly L., Casey B., Gelius P., Messing S., Forberger S., Lakerveld J., Zukowska J., Bengoechea E.G. (2021). The Evidence for the Impact of Policy on Physical Activity Outcomes within the School Setting: A Systematic Review. J. Sport Health Sci..

[24] Jones M., Defever E., Letsinger A., Steele J., Mackintosh K.A. (2020). A Mixed-Studies Systematic Review and Meta-Analysis of School-Based Interventions to Promote Physical Activity and/or Reduce Sedentary Time in Children. J. Sport Health Sci..

[25] Tremblay M.S., Carson V., Chaput J.-P., Connor Gorber S., Dinh T., Duggan M., Faulkner G., Gray C.E., Gruber R., Janson K. (2016). Canadian 24-Hour Movement Guidelines for Children and Youth: An Integration of Physical Activity, Sedentary Behaviour, and Sleep. Appl. Physiol. Nutr. Metab..

[26] Piercy K.L., Troiano R.P., Ballard R.M., Carlson S.A., Fulton J.E., Galuska D.A., George S.M., Olson R.D. (2018). The Physical Activity Guidelines for Americans. JAMA.

[27] Alcántara-Porcuna V., Sánchez-López M., Martínez-Vizcaíno V., Martínez-Andrés M., Ruiz-Hermosa A., Rodríguez-Martín B. (2021). Parents’ Perceptions on Barriers and Facilitators of Physical Activity among Schoolchildren: A Qualitative Study. Int. J. Environ. Res. Public Health.

[28] Guevara R.M., Urchaga J.D., Cabaco A.S., Moral-García J.E. (2020). The Quality of Breakfast and Healthy Diet in School-Aged Adolescents and Their Association with BMI, Weight Loss Diets and the Practice of Physical Activity. Nutrients.

[29] Cimminiello C., Vlachopulos C., De Carlo M. (2021). The 2021 ESC Guidelines on Cardiovascular Prevention: Whether the Ends Justify the Means. Eur. J. Intern. Med..

[30] Visseren F.L., Mach F., Smulders Y.M., Carballo D., Koskinas K.C., Bäck M., Benetos A., Biffi A., Boavida J.-M., Capodanno D. (2021). 2021 ESC Guidelines on Cardiovascular Disease Prevention in Clinical Practice. Eur. Heart J..

